# Determinants of Growth in Prescription Drug Spending Using 2010–2019 Health Insurance Claims Data

**DOI:** 10.3389/fphar.2021.681492

**Published:** 2021-05-31

**Authors:** HyeYeong Lee, Dahye Park, Dong-Sook Kim

**Affiliations:** Department of Research, Health Insurance Review and Assessment Service, Wonju, South Korea

**Keywords:** drug spending, price, quantity, determinants, claims database

## Abstract

**Background:** Despite policies to manage prescription drug spending and ensure accessibility, prescription drug spending has continued to increase in South Korea. Using nationwide claims data, this study analyzed trends in total pharmaceutical expenditures and pharmaceutical expenditures by drug classification.

**Methods:** We conducted a retrospective population-based study using the Korean National Health Insurance claims database from January 2010 through December 2019. Pharmaceuticals were categorized as new drugs, continued drugs, and abandoned drugs. Prescription drug spending was calculated using the components of price and quantity for individual products in successive two-year periods, to obviate the need to consider changes over time.

**Results:** Total pharmaceutical expenditures increased by 54.2% from 2010 to 2019 (from USD 11.3 billion to USD 17.4 billion). The average annual growth rate was 4.9% overall (the 4% rate for continued drugs was decomposed into −3.5% for the price of drugs, 8.0% for the quantity of drugs, and −0.5% for mixed effects, a measure of changes in drug treatment patterns). The trends were generally consistent. Particularly sharp increases in expenditures were found for groups L (antineoplastic and immunomodulating agents), C (cardiovascular system drugs), and A (alimentary tract and metabolism drugs).

**Conclusions:** Since increased prescription drug spending was primarily driven by an increase in the quantity of drugs used, consumer-focused policies to reduce drug use are necessary.

## Introduction

In many countries, healthcare expenditures after the national health insurance system implemented medication coverage have increased faster than GDP and total public expenditures (OECD, 2020). In particular, with the introduction of direct acting antiviral agents for hepatitis C, expensive medications for rare diseases, expensive anticancer drugs, and individualized medications, the financial sustainability of health systems is being threatened (OECD, 2019). Therefore, in most countries, policies have been implemented to curb prescription drug spending, but prescription drug spending remains a challenging issue (Belloni et al., 2016).

South Korea became an aging society in 2000 and an aged society in 2018. It is expected that in 2025, Korea will become a super-aged society, with 20% of the population exceeding 65 years of age (KOSIS, 2019). The Korean government introduced the National Health Insurance (NHI) system in 1989. The NHI currently covers 97% of the population, while the remaining 3% are covered by the Medical Aid Program. Korea has achieved universal health insurance with a fee-for-service system. The list of medicines reimbursable under the NHI is uniformly applied nationwide. The Ministry of Health and Welfare (MOHW) and Health Insurance Review and Assessment Service (HIRA) determine pharmaceutical reimbursements based on a value assessment (health technology assessment, HTA) considering clinical usefulness and an economic evaluation. After unifying several payers into a single payer in 2000, the health insurance system faced issues with fiscal deficits in the early 2000s. Thus, many policies aiming to limit medical and prescription drug spending have been implemented. On the supply side, the positive listing system was implemented in 2007, drug price reduction from 2012 to 2013, price–volume agreement in January 2014, and drug price reduction based on market transaction survey results in March 2016 (Kwon et al., 2015). On the demand side, reviews of doctors’ prescription patterns, incentives to reduce prescription drug spending, alternative preparations by pharmacists, patient out-of-pocket payments, and diagnosis-related groups in hospitals were implemented. Simultaneously, in order to improve patients’ access to medicines, policies were implemented to expand health insurance coverage. The Korean government reduced patients’ co-payments of total healthcare expenditures for four major diseases (cancer, rare diseases, cardiovascular diseases, and cerebrovascular diseases) in 2013 and all diseases in 2018, alleviated decision-making of reimbursement for new medicines, and expanded the scope of benefits, such as other indications of already listed drugs. To increase access to new medicines and reduce the financial burden of health insurance, risk-sharing arrangements between government and pharmaceutical companies have also been introduced since 2013 (Kwon and Godman, 2017).

Rising prescription drug spending has remained a constant issue in South Korea. As a benchmark for comparison, the rate of increase in prescription drug spending in Organization for Economic Co-operation and Development (OECD) countries was 1.6% from 2013 to 2017, but the rate of increase in expenditures was 4.2% for retail pharmaceuticals and 7.9% for hospital pharmaceuticals in 2017 (OECD, 2020). Due to reduced insurance premium income from an aging population and expected economic difficulties from the expansion of health insurance coverage, the financial burden on South Korea’s national insurance system is concerning.

It is expected that expensive new drugs will continue to be introduced, so it is necessary for policy-makers to understand factors that drive growth in prescription drug spending and promote efficiency in financial expenditures. Several studies have classified the drivers of increasing drug spending into price, quantity (number of prescriptions), and mixed effects (Gerdtham, 1993; Gerdtham et al., 1998; Dubois et al., 2000; Chernew et al., 2001; Aitken et al., 2009). Mixed effects refer to changes in the types of drugs used for the same injury or disease in the same treatment class (OECD, 2019). However, few studies have examined changes in price and quantity by drug therapeutic classification. Therefore, this study aimed to measure the effects of price and quantity on prescription drug spending from 2010 to 2019 in South Korea.

## Methods

### Data Source and Categories

We conducted a retrospective population-based study using the Korean National Health Insurance claims database from January 2010 through December 2019. This database contained information on both in-hospital and outpatient visits from a population of 51.8 million as of 2020. The database includes demographic characteristics, diagnosis, healthcare utilization (visit date, test, procedures, length, and spending), and medicine use (product name, ingredient name, dose, days of therapy, and spending). All claims have been submitted electronically since 2007, and all data files (e.g., type of medical facilities and patients’ demographic files) could be linked by unique patient identification numbers.

The analytical unit of this study was medication (different active ingredients and doses). Prescription drug spending referred to total spending, including patient out-of-pocket fees and value-added tax (VAT); this was calculated from the medications listed in the healthcare claims data from the entire population.

We analyzed changes in prescription drug spending according to each dimension using the health insurance database. The analytical dimensions were inpatient/outpatient, type of medical institution, and drug classification. The therapeutic classification followed the World Health Organization Anatomical Therapeutic Chemical (ATC) system (WHOCC, 2020).

Pharmaceuticals were categorized into new drugs, continued drugs, and abandoned drugs. New chemical entities were defined as products manufactured by a single company with an active ingredient that was newly listed in the cumulative health insurance reimbursement list of medicines that year and not claimed in any previous years. Continued drugs were drugs that were included in the reimbursement list and used in a given year and the previous year, and abandoned drugs were drugs that were not used after a given year.

### Trend of Healthcare Spending

Trend analysis was conducted using prescription spending, macroscopic indicators such as economic and demographic changes, and the number of listed medicines in health insurance benefit coverage. We collected data on GDP per capita, medical expenditures as a proportion of GDP, and pharmaceutical expenditures as a proportion of medical expenditures from the OECD, and gathered data on the total population and the population above 65 years of age from the Korean Statistical Information Service (KOSIS) for each year.

We analyzed the number of drugs (new drugs, continued drugs, and abandoned drugs), total pharmaceutical expenditures, and pharmaceutical spending per patient by year. We also determined total monthly pharmaceutical spending overall and by sector.

### Determinants of Pharmaceutical Spending and Analysis

We analyzed the categories of new drugs, continued drugs, and abandoned drugs. During the 10-year study period, the increase from previous years was calculated using two-year intervals. Prescription drug spending was calculated using the components of price and quantity by individual products. The decomposition equation was as follows. The mixed effect was analyzed by the composition ratio of the main components.

Expenditure (E)=Price (p)×Mixed effect×Quantity(Q), (1)

$$\frac{E_{T^{1}}}{E_{T^{0}}}-1=\{\theta^{E_{C}}\times \left(\frac{E_{C^{1}}}{E_{C^{0}}}-1\right)\}+\{\theta^{E_{A}}\times \left(-1\right)\}+\frac{E_{N^{1}}}{E_{T^{0}}}$$ (2)

where $E_{T^{0}}$ is the total prescription drug spending in the previous year, $E_{T^{1}}$ is the total prescription drug spending in the given year, $E_{C^{0}}$ is the continued drug spending in the previous year, $E_{C^{1}}$ is the continued drug spending in the given year, $E_{N^{1}}$ is the new drug spending in the given year, $E_{A^{0}}$ is the abandoned drug spending in the previous year, $\theta^{E_{C}}$, $\left(E_{C^{0}}/E_{T^{0}}\right)$ is the share of continued drug spending in the previous year, and $\theta^{E_{A}}$, $(E_{A^{0}}/E_{T^{0}})$ is the share of discontinued or abandoned drug spending in the previous year.

Newly listed drugs were chemical substances/doses that were utilized for the first time in a given year and not listed until that year. Although no information was available for quantity in the previous year for new drugs and in the next year for abandoned drugs, we calculated both the price and quantity for continued drugs. In order to examine changes in the price and quantity in continued drugs, a price index and a quantity index for each time period were calculated for continued drug spending. Quantity was defined as the length of prescription. Quantity was subdivided into quantity by the main ingredient and quantity by drug classification (ATC level 3).

To calculate the indices, the Fisher index, which allocates the same weight to the reference time point and the comparison time point, was used (Fisher, 1922). The Fisher ideal index $\left(I^{F}=\sqrt{I^{l}\times I^{P}}\right)$ is the geometric mean of the Laspeyres index and the Paasche index (Hoekstra et al., 2003), defined as follows:

$$I^{P}\left(\text{Price index}\right)=\sqrt{\frac{\sum_{i}^{m}\sum_{j}^{n_{i}}p_{1ij}\times u_{0ij}\times q_{0iㆍ}}{\sum_{i}^{m}\sum_{j}^{n_{i}}p_{0ij}\times u_{0ij}\times q_{0iㆍ}}\times \frac{\sum_{i}^{m}\sum_{j}^{n_{i}}p_{1ij}\times u_{1ij}\times q_{1iㆍ}}{\sum_{i}^{m}\sum_{j}^{n_{i}}p_{0ij}\times u_{1ij}\times q_{1iㆍ}}},$$ (3)

$$I^{U}\left(\text{Composition index}\right)=\sqrt{\frac{\sum_{i}^{m}\sum_{j}^{n_{i}}p_{0ij}\times u_{1ij}\times q_{0iㆍ}}{\sum_{i}^{m}\sum_{j}^{n_{i}}p_{0ij}\times u_{0ij}\times q_{0iㆍ}}\times \frac{\sum_{i}^{m}\sum_{j}^{n_{i}}p_{1ij}\times u_{1ij}\times q_{1iㆍ}}{\sum_{i}^{m}\sum_{j}^{n_{i}}p_{1ij}\times u_{0ij}\times q_{1iㆍ}}},$$ (4)

$$I^{Q}\left(\text{Quantity index}\right)=\left(\frac{E_{C^{1}}/E_{C^{0}}}{I^{P}\times I^{U}}\right)$$ (5)

where *p*_0*ij*_ is the price of the *j*th item in the *i*th main ingredient group in the previous year, *p*_1*ij*_ is the price of the *j*th item in the *i*th main ingredient group in the given year, *u*_0*ij*_ is the share of the *j*th item in the *i*th main ingredient group in the previous year, *u*_1*ij*_ is the share of the *j*th item in the *i*th main ingredient group in the given year (=share by item), *q*_0*i*_ is the total quantity of *n*_*i*_ items that have the *i*th main ingredient in the previous year, and *q*_1*i*_ is the total quantity of *n*_*i*_ items that have the *i*th main ingredient in the previous year (=total quantity by main ingredient).

The price factor used in the price index was the price per day of the prescription, and the corresponding quantity factor was the length of the prescription. When the composition ratio used to calculate the price index, quantity index, and composition index in the Fisher index was set as the composition within the same main ingredient group, the quantity factor was calculated as quantity by the main ingredient. The list and real prices are almost the same in Korea, and we used the real price from the given year. Although 10 years is quite a long time, we compared expenditures for successive two-year periods to obviate the need to consider inflation. The price index measured the effect of price changes on the increase in prescription drug spending, as the quantity and share in the main ingredient group were fixed at the comparison time point. The composition ratio measured the shift from low-price pharmaceuticals to high-price pharmaceuticals. The impact of the composition ratio on the increase in prescription drug spending was measured by fixing the price and quantity while changing the weight within the main ingredient group.

$$\begin{array}{l} \text{Prescription drug spending}\left(E_{C}\right)=\;\text{Price per drug}\left(\text{p}\right)\times \text{Composition ratio within the main ingredient group }\left(\text{MIX}\right),\\ \times \text{ Quantity by main ingredient }\left(Q\right)=\sum_{i=1}^{m}\sum_{j=1}^{n_{i}}\left[p_{ij}\times \frac{q_{ij}}{\sum_{j}^{n_{i}}q_{ij}}\times \sum_{j}^{n_{i}}q_{ij}\right] \end{array}$$ (5)

where *n*_*i*_ is the number of items in the *i*th main ingredient group, *p*_*ij*_ is the price of the *j*th item in the *i*th main ingredient group (= price per day of prescription), $\frac{q_{ij}}{\sum_{j}^{n_{i}}q_{ij}}$ is the share of the *j*th item in the *i*th main ingredient group, and $\sum_{j}^{n_{i}}q_{ij}$ is the total quantity of *n*_*i*_ items that contain the *i*th main ingredient (= total length of prescription).

## Results

### Pharmaceutical Expenditures Overall and by Sector

The total population and the population above 65 years of age increased. The proportion of elderly adults increased from 10.7% in 2010 to 15.1% in 2019. GDP per capita, as an economic indicator, increased steadily. Medical expenditures as a proportion of GDP also increased steadily from 5.9% in 2010 to 8.0% in 2019. In contrast, pharmaceutical expenditures as a proportion of medical expenditures decreased steadily from 24.7% in 2010 to 20.0% in 2019.

Total pharmaceutical expenditures increased from USD 11.3 billion in 2010 to USD 17.4 billion in 2019, representing a 54.2% increase. Total healthcare expenditures increased significantly with the introduction of new healthcare diagnostic technology; as a result, pharmaceutical expenditures as a proportion of medical expenditure decreased. The absolute amount of pharmaceutical expenditures decreased until April 2012 and increased thereafter. The average increase per year was 4.9%. Pharmaceutical expenditures decreased due to the drug price reduction policy from 2012 to 2013, but steadily increased since 2014. After the expansion policy of health insurance coverage in 2016, total pharmaceutical expenditures increased. Pharmaceutical expenditures per person older than 65 years increased from USD 0.77 thousand in 2010 to USD 0.96 thousand in 2019, corresponding to an average annual growth rate per year of 2.51%. In contrast, the average annual growth rate of pharmaceutical expenditures per person under 65 years was 3.65% (Table 1).

**TABLE 1:** Trends in pharmaceutical expenditures and economic and demographic changes by year.

	2010	2011	2012	2013	2014	2015	2016	2017	2018	2019	Change (CAGR) (%)
**Demographic changes** ^a^											
Total population (1,000 population)	49,554	49,937	50,200	50,429	50,747	51,015	51,218	51,362	51,607	51,709	0.47
Population above 65 years of age (1,000 population 65+)	5,288	5,516	5,795	6,057	6,319	6,566	6,790	7,148	7,459	7,826	4.45
Proportion of elderly	10.7%	11.0%	11.5%	12.0%	12.5%	12.9%	13.3%	13.9%	14.5%	15.1%	3.90
**Expenditures**											
Gross domestic product (GDP) per capita (USD)^b^	23,083	25,100	25,458	27,178	29,242	28,724	29,287	31,605	33,429	31,838	3.64
Total medical expenditures as a proportion of GDP^b^	5.9%	6.0%	6.1%	6.2%	6.5%	6.7%	6.9%	7.1%	7.6%	8.0%	3.44
Total medical expenditures (billion USD)^c^	65	70	74	78	84	92	100	109	119	128	7.82
Total pharmaceutical expenditures (billion USD)	11.3	11.8	11.4	11.6	12.1	12.7	14.0	14.8	16.1	17.4	4.91
Total pharmaceutical expenditures per person (thousand USD, under 65)	0.16	0.17	0.16	0.16	0.17	0.17	0.19	0.20	0.21	0.22	3.65
Total pharmaceutical expenditures per person (thousand USD, 65+)	0.77	0.79	0.74	0.74	0.75	0.77	0.83	0.86	0.91	0.96	2.51
Pharmaceutical expenditures as a proportion of medical expenditures	24.7%	24.2%	23.3%	22.2%	21.4%	20.8%	20.8%	20.4%	20.0%	20.0%	−2.32
**Number of medicines**											
Total number of products	14,169	13,663	13,580	14,568	15,656	17,361	19,817	20,636	21,134	21,160	4.56
Number of new drugs	–	198	238	259	254	488	395	324	275	232	2.00
Number of continued drugs	13,921	14,715	14,163	14,784	15,860	17,256	19,830	21,054	21,651	22,659	5.56
Number of abandoned drugs	248	248	271	329	228	229	186	372	266	399	5.43

a Korean Statistical Information Service (KOSIS) census data.

b OECD.

c Korean Statistical Information Service, update 09-08-2020 (MDY).

CAGR, compound annual growth rate.

**FIGURE 1:**
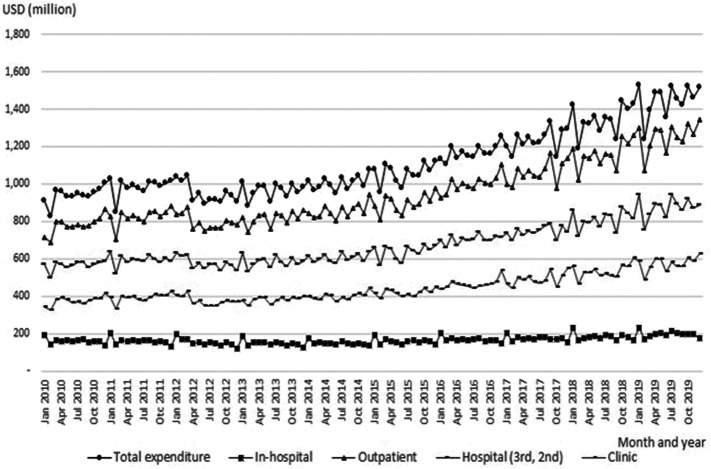
Monthly pharmaceutical expenditures (total and by sector).

Figure 1 presents the monthly pharmaceutical expenditures, overall, and by sector, between 2010 and 2019. In certain months, expenditures decreased when fewer outpatient visits were made due to holidays. There were major increases in expenditures in the outpatient sector in 2016, 2018, and 2019. Pharmaceutical expenditures jumped in secondary and tertiary hospitals in 2016, 2018, and 2019.

### Determinants of Pharmaceutical Expenditures

Table 2 examines the components of total pharmaceutical expenditures (price, quantity, and mixed effects) by categories of drugs (new, continued, and abandoned) and type of healthcare utilization (inpatient or outpatient).

**TABLE 2:** Contributions to changes in pharmaceutical spending by 2-year periods, overall and by sector (%).

	2010–2011 vs. 2012–2013	2012–2013 vs. 2014–2015	2014–2015 vs. 2016–2017	2016–2017 vs. 2018–2019	Annual average	(Change)
Total Relative increment	−0.74	7.82	16.40	16.09	4.9	(100)
New drugs	1.58	2.19	2.69	1.70	1.0	(21)
Continued drugs	−2.22	5.81	13.77	14.67	4.0	(81)
Price (P)	−16.34	−6.51	−2.19	−3.04	−3.5	(−71)
Mixed effect in the main active ingredient	−1.36	−1.24	−1.09	−0.22	−0.5	(−10)
Quantity (Q)	15.48	13.56	17.05	17.92	8	(162)
Abandoned drugs	−0.10	−0.17	−0.06	−0.27	−0.1	(−2)
Inpatient relative increment	−6.05	1.92	10.77	10.89	2.2	(100)
New drugs	0.62	3.27	1.68	2.64	1.0	(47)
Continued drugs	−6.48	−1.16	9.21	8.48	1.3	(57)
Price (P)	−15.62	−7.45	−4.14	−3.58	−3.8	(−176)
Mixed effect in the main active ingredient	−0.67	−0.45	−0.93	0.01	−0.3	(−12)
Quantity (Q)	9.81	6.74	14.28	12.05	5.4	(245)
Abandoned drugs	−0.19	−0.19	−0.12	−0.23	−0.1	(−4)
Outpatient Relative increment	0.31	8.91	17.38	16.94	5.4	(100)
New drugs	2.09	2.01	4.61	1.56	1.3	(24)
Continued drugs	−1.58	7.10	12.82	15.69	4.3	(78)
Price (P)	−16.80	−6.31	−1.58	−2.94	−3.5	(−63)
Mixed effect in the main active ingredient	−1.50	−1.40	−1.19	−0.24	−0.5	(−10)
Quantity (Q)	16.72	14.81	15.59	18.87	8.2	(152)
Abandoned drugs	−0.20	−0.21	−0.05	−0.31	−0.1	(−2)

Changes were calculated as the annual average.

The average rate of increase per year was 4.9%, and it was divided into 1% for new drugs, 4% for continued drugs, and -0.1% for abandoned drugs. For continued drugs, we decomposed the trend into –3.5% for price, 8.0% for quantity, and –0.5% for mixed effects. The contribution of product price steadily showed the effect of reducing drug costs for continued drugs. The only decrease in pharmaceutical expenditures was observed after the reduction in all drug prices in 2012, and the magnitude of the reduction was slight. Although drug prices continued to decrease, the quantity of high-price drugs used steadily increased since 2014, eventually accounting for the largest proportion among the components of pharmaceutical expenditures.

### Contribution by Drug Classification

**FIGURE 2:**
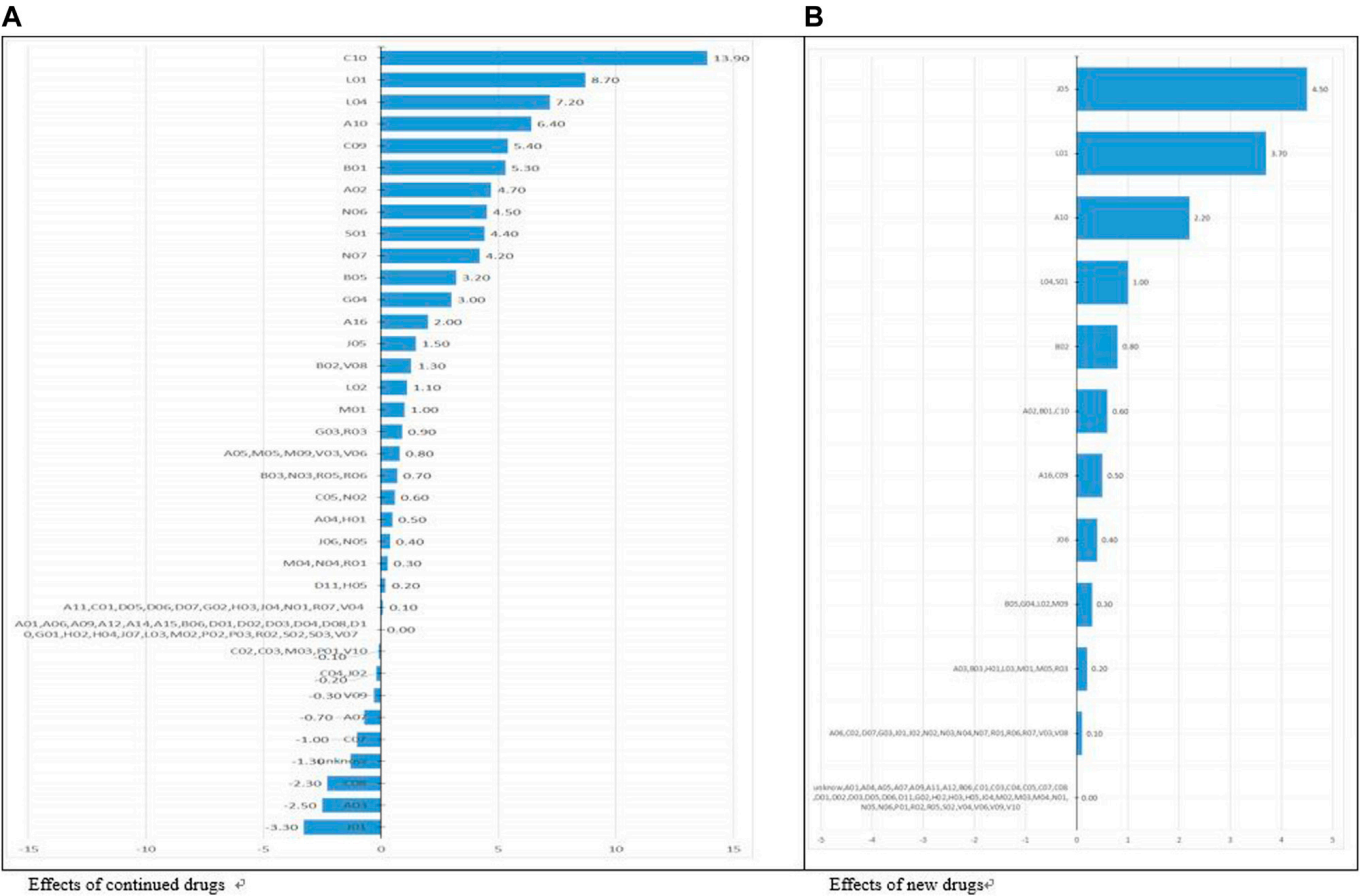
Effects of drugs by categories (continued, new) categorized by ATC level 2 (%).

Table 3 shows changes in and contributions to pharmaceutical expenditures (divided into continued, new, and other drugs) by drug classification. Among continued drugs, changes in and contributions to pharmaceutical expenditures increased for most drug groups. Classified using ATC level 1, a large increase was seen for continued and new drugs in groups L (antineoplastic and immunomodulating agents), C (cardiovascular system), and A (alimentary tract and metabolism). When drugs were classified using ATC level 2, the highest increase in total average pharmaceutical expenditures per year was found for C10 (lipid modifying agents), followed by L01 (antineoplastic agents). In ATC level 3, the highest expenditures were found for L01X (other antineoplastic agents), C10 A (lipid modifying agents, plain), and L04 A (immunosuppressants), in a descending order. Among new drugs, the highest annual average increases, and changes were found for groups L (antineoplastic and immunomodulating agents) and J (anti-infective for systemic use) in the ATC level 1 classification. Using ATC level 2, the highest values were found for L04 (immunosuppressants), and using ATC level 3, the highest values were found for J05 A (direct acting antivirals), L01X (other antineoplastic agents), and A10 B (blood glucose lowering drugs, excluding insulins) in a descending order (Figure 2). The ingredients with the largest annual growth rate among the continued products were rosuvastatin and ezetimibe (8%), followed by osimertinib (5%), atorvastatin (4%), rosuvastatin (4%), pembrolizumab (4%), and choline alfoscerate (4%). For new drugs, the highest annual growth rate was found for sofosbuvir (2%).

**TABLE 3:** Contributions to changes in pharmaceutical spending by two-year periods for ATC level 1 groups of medicines (%).

	2010–2011 vs. 2012–2013			2012–2013 vs. 2014–2015			2014–2015 vs. 2016–2017			2016–2017 vs. 2018–2019			Annual average			(Change)		
	N	C	A	N	C	A	N	C	A	N	C	A	N	C	A	N	C	A
Total	1.58	−2.22	−0.10	2.19	5.81	−0.17	2.69	13.77	−0.06	1.70	14.67	−0.27	1.0	4.0	−0.1	(21)	(81)	(−2)
A	0.44	−1.27	−0.03	0.43	1.20	−0.13	0.37	2.37	0.00	0.19	2.14	−0.01	0.2	0.6	−0.02	(4)	(11)	(−0.4)
B	0.12	0.09	0.00	0.17	1.02	0.00	0.37	1.55	−0.03	0.10	1.55	−0.09	0.1	0.5	−0.02	(2)	(11)	(−0.3)
C	0.12	0.06	−0.01	0.14	0.73	0.00	0.15	2.59	0.00	0.07	3.11	0.00	0.1	0.8	0.00	(1)	(16)	(0.0)
D	0.01	−0.07	0.00	0.01	−0.11	0.00	0.04	0.43	0.00	0.01	−0.09	−0.02	0.0	0.0	0.00	(0)	(0)	(−0.1)
G	0.06	0.05	0.00	0.03	0.52	0.00	0.07	0.35	0.00	0.02	0.63	0.00	0.0	0.2	0.00	(0)	(4)	(0.0)
H	0.00	0.09	0.00	0.01	0.01	0.00	0.01	0.11	0.00	0.06	0.12	0.00	0.0	0.0	0.00	(0)	(1)	(0.0)
J	0.27	−1.21	0.00	0.18	0.45	0.00	1.14	0.57	0.00	0.41	−0.46	−0.03	0.3	−0.1	0.00	(5)	(−2)	(−0.1)
L	0.25	0.75	0.00	0.86	0.64	0.00	0.35	2.09	0.00	0.60	3.28	−0.05	0.3	0.8	−0.01	(5)	(17)	(−0.1)
M	0.09	−0.62	0.00	0.07	0.25	−0.01	0.03	0.64	0.00	0.11	0.84	0.00	0.0	0.1	0.00	(1)	(3)	(0.0)
N	0.02	0.24	−0.01	0.04	0.76	0.00	0.03	1.37	0.00	0.06	1.90	0.00	0.0	0.5	0.00	(0)	(11)	(0.0)
*P*	0.00	−0.02	0.00	0.00	0.00	0.00		0.00			0.00	0.00	0.0	0.0	0.00	(0)	(0)	(0.0)
R	0.05	−0.22	0.00	0.09	0.26	0.00	0.04	0.50	0.00	0.02	0.53	−0.02	0.0	0.1	0.00	(1)	(3)	(−0.1)
S	0.12	−0.09	0.00	0.15	0.22	0.00	0.07	0.77	0.00	0.05	0.86	−0.01	0.0	0.2	0.00	(1)	(5)	(0.0)
V	0.02	0.33	0.00	0.02	0.03	0.00	0.02	0.43	0.00	0.01	0.26	−0.03	0.0	0.1	0.00	(0)	(3)	(−0.1)

Changes were calculated as the annual average (based on 7 decimal points).

N, new drugs; C, continued drugs; A, abandoned drugs.

A, Alimentary Tract and Metabolism.

B, blood and blood-forming organs.

C, cardiovascular system.

G, dermatologicals.

H, genitourinary system and sex hormones.

J, systemic hormonal preparations, Excl. sex hormones, and insulins.

M, anti-infectives for systemic use.

N, antineoplastic and immunomodulating agents.

R, musculoskeletal system.

D, nervous system.

L, antiparasitic products, insecticides, and repellents.

*p*, respiratory system.

S, sensory organs.

V, various.

Table 4 demonstrates the continued drugs that had the largest impact on the rate of increase in pharmaceutical expenditures (divided into the components of price, quantity, and mixed effects) by drug classification. Similar to Table 3, anomalous trends were only observed from 2012 to 2013, when the drug price reduction policy was implemented, and the trend was similar in other years. ATC 1-level L (antineoplastic and immunomodulating agents) showed a large increase in price. While the prices of groups A (alimentary tract and metabolism) and J (anti-infective for systemic use) were relatively low, the increase in the quantity used was high.

**TABLE 4:** Pharmaceutical expenditures of continued drugs by 12 ATC 1-level groups during the period 2010–2019 (%).

	2010–2011 vs. 2012–2013			2012–2013 vs. 2014–2015			2014–2015 vs. 2016–2017			2016–2017 vs. 2018–2019			Annual average			(Change)		
	Price	Quantity	Mixed	Price	Quantity	Mixed	Price	Quantity	Mixed	Price	Quantity	Mixed	Price	Quantity	Mixed	Price	Quantity	Mixed
Total	−16.34	21.66	−7.55	−6.51	10.69	1.63	−2.19	8.21	7.74	−3.04	11.22	6.48	−3.5	6.5	1.0	(−71)	(131)	(21)
A	−9.30	−1.35	−0.38	−0.44	8.81	2.81	2.93	2.61	−0.78	−0.26	−0.19	−0.03	−1.4	2.1	−0.2	−29	43	−3
B	0.67	−1.14	−0.25	−0.32	−0.64	2.38	1.92	1.89	0.06	−0.22	−0.12	−0.02	−0.1	0.7	0.0	−3	14	−1
C	0.44	−0.82	−0.41	−0.64	−0.41	1.72	3.20	3.80	0.04	−0.16	−0.20	−0.05	−0.2	1.0	0.0	−4	21	−1
D	−0.51	0.12	−0.07	0.02	0.48	−0.25	0.54	−0.11	−0.04	0.02	−0.03	0.00	−0.1	0.1	0.0	−1	2	0
G	0.34	−0.58	−0.06	−0.13	−0.32	1.21	0.44	0.77	0.03	−0.11	−0.03	−0.01	−0.1	0.3	0.0	−1	5	0
H	0.66	−0.02	−0.02	−0.02	−0.63	0.03	0.14	0.14	0.06	0.00	−0.01	0.00	0.1	0.0	0.0	2	−1	0
J	−8.89	−0.50	−0.09	0.10	8.42	1.05	0.71	−0.56	−0.74	−0.10	−0.05	0.01	−1.2	1.2	−0.1	−24	24	−2
L	5.51	−0.72	−0.33	−0.68	−5.22	1.50	2.58	4.00	0.46	−0.14	−0.17	−0.05	0.5	0.4	0.0	10	7	0
M	−4.59	−0.28	−0.10	−0.17	4.34	0.58	0.79	1.03	−0.38	−0.05	−0.05	−0.01	−0.6	0.8	−0.1	−13	17	−1
N	1.77	−0.86	−0.22	−0.39	−1.67	1.79	1.69	2.32	0.15	−0.16	−0.11	−0.03	0.0	0.5	0.0	1	10	0
P	−0.12	0.00	0.00	0.00	0.11	−0.01	0.00	0.00	−0.01	0.00	0.00	0.00	0.0	0.0	0.0	0	0	0
R	−1.65	−0.29	−0.08	−0.11	1.56	0.60	0.62	0.65	−0.14	−0.06	−0.04	−0.01	−0.3	0.4	0.0	−5	9	−1
S	−0.68	−0.25	−0.12	−0.18	0.65	0.52	0.96	1.05	−0.06	−0.05	−0.06	−0.01	−0.2	0.4	0.0	−3	8	−1
V	2.40	−0.03	−0.07	−0.05	−2.27	0.07	0.53	0.32	0.20	−0.01	−0.03	0.00	0.3	−0.2	0.0	6	−3	0

Notes: Changes were calculated as the annual average (based on 7 decimal points).

A, Alimentary Tract and Metabolism.

B, blood and blood-forming organs.

C, cardiovascular system.

G, dermatologicals.

H, genitourinary system and sex hormones.

J, systemic hormonal preparations, excl. sex hormones, and insulins.

M, anti-infectives for systemic use.

N, antineoplastic and immunomodulating agents.

R, musculoskeletal system.

D, nervous system.

L, antiparasitic products, insecticides, and repellents.

*p*, respiratory system.

S, sensory organs.

V, various.

## Discussion

This study measured the effects of price and quantity on prescription drug spending from 2010 to 2019 in South Korea by dimension and drug classification. Using the average rate of increase in total prescription drug spending over the 10 years of 4.9% as a reference value (100%), the relative increase was 81% for continued drugs, 21% for new drugs, and –2% for abandoned drugs. The relative change in the price factor was –71% on average, that of the mixed factor of price and quantity was −10%, and that of the quantity factor was 162%.

We found a decreasing trend in price and an increasing trend in quantity, and this result is similar to that of the previous research that the change of prices had a decreasing effect on drug expenditures (Kwon et al., 2015; Jo et al., 2016). This finding appears to be the result of continued policy efforts to reduce drug prices. The use of newer and more expensive products has also been identified in previous studies as a significant cost driver in many drug classes (Gerdtham and Lundin, 2004; Morgan et al., 2004; Soppi et al., 2018). Our study showed that continued drugs had the largest impact on the growth in pharmaceutical expenditures, followed by new drugs and abandoned drugs, in a descending order.

Among the continued products, mixed effects were found due to changes in treatment trends. This finding is in accordance with results reported in previous studies. The mixed effects had diverging trends in inpatient and outpatient settings. For inpatients, the rate of change was −12%, indicating a shift to lower-price drugs, and for outpatients, the rate was 10%, indicating a shift to higher-price drugs. Previous research found that the quantity of drugs contributed to increased prescription drug spending (Jo et al., 2016; Kwon, Kim, et al., 2015). Jo et al. analyzed data from June 2012 to 2018 and reported that mixed effects accounted for 40–60% within the drug classification category and 30–40% within ingredients (Jo et al., 2016). According to this study, the total mixed effect increased by 21%, while it decreased for inpatients. The contributors to increased prescription drug spending were found to be different by sector. However, quantity increased sharply for both inpatients and outpatients.

By drug classification, the greatest increase was found in groups L, C, and A of ATC level 1. Due to the influx of new medications, spending for antineoplastic and immunomodulating agents (L) such as anticancer medications, anti-infectives for systemic use (J) such as antivirus drugs, and alimentary tract and metabolism (A) such as diabetes medication increased. Among continued medications, spending for antineoplastic and immunomodulating agents (L) such as anticancer medications and cardiovascular system (C) such as antilipidemic drugs increased. However, the prices of alimentary tract and metabolism (A) such as diabetes medication and anti-infectives for systemic use (J) such as antibiotics decreased, but the quantity increased. The mixed effect showed that treatment trends shifted to expensive antineoplastic and immunomodulating agents (L) such as anticancer medications.

Although the absolute amount of pharmaceutical expenditures increased, pharmaceutical expenditures as a proportion of total medical expenditures decreased because total medical spending increased more significantly with the introduction of new diagnostic technology. Moreover, it seems that the ongoing implementation of cost containment policies for pharmaceutical expenditures has stabilized pharmaceutical spending. We hypothesized that aging might drive growth in the quantity of drugs; however, the increase in pharmaceutical expenditures in individuals over the age of 65 years was lower than that in under-65 individuals. This means that the increase in prescription drug quantity and spending was due to changes in treatment patterns or diagnostic technology rather than aging. According to the OECD report “Tackling wasteful spending on health,” nearly 20–33% of total health expenditures could be deemed wasteful (OECD, 2017). The OECD report stated that low-value care includes over-testing, unnecessary surgical interventions, and the inappropriate use of antimicrobials. To reduce inappropriate use and waste, the OECD suggested that interventions such as performance- and value-based payments, and patient co-payments for low-value care should be introduced (OECD, 2017).

To our knowledge, this is the first study that analyzed the contributors to increased prescription drug spending in the past 10 years in a representative manner. Thus, the results are generalizable. Second, considering that the total study duration (10 years) is a long period, it is meaningful that new, continued, and abandoned drugs were analyzed at two-year intervals, resulting in analytic units of two-year periods, to analyze the effect of each type on increased prescription drug spending. Third, differences in price, quantity, and mixed effects within the drug classification categories were observed. Fourth, the analysis by institution type (inpatient and outpatient) was also a strength. Last, most existing studies discussed contributors to prescription drug spending in terms of price, quantity, and mixed effects, but only one study examined the drug classification. This study explored contributions to recent increases in prescription drug spending by drug classification. However, this study did not consider the daily dose when calculating quantity, since price was defined as the price per day of the prescription and quantity was defined as the length of the prescription. Policy-makers and national insurance administrators should focus more on managing quantity and mixed effects when managing prescription drug spending.

Our study also has several limitations. First, our analysis examined trends due to population aging, but did not correct for this factor by adjusting for patient composition. The increase in the number of elderly population and individuals with chronic diseases is likely to have affected spending significantly. Second, changes in the economy, policies, and system beyond supply and demand aspects may have had a considerable influence on prescription drug spending. The reduction in drug prices that occurred from 2012 to 2014 may have had a major effect on the drop in prescription drug expenditures during that period. The analysis took this timing into consideration, but it was not possible to account for all other policy changes. Third, when calculating prescription drug spending, preparation fees were not included in this study.

In conclusion, after decomposing the increase in prescription drug spending, it was found that the increase was primarily driven by the quantity of continued drugs, so policies to address this issue should be prepared. These are important concerns for a policy when establishing the pharmaceutical policies for rational volume control of continued drugs and deliberate decision-making for the reimbursement of new medicines. Further study is also needed on strategies to reduce the volume of inappropriate use of low-value care.

## Data Availability

The datasets presented in this article are not readily available because access to the dataset is limited. Request to access the datasets should be directed to our institution.
